# Spatial Dependency of Tuberculosis Incidence in Taiwan

**DOI:** 10.1371/journal.pone.0050740

**Published:** 2012-11-30

**Authors:** In-Chan Ng, Tzai-Hung Wen, Jann-Yuan Wang, Chi-Tai Fang

**Affiliations:** 1 Institute of Epidemiology and Preventive Medicine, College of Public Health, National Taiwan University, Taipei, Taiwan; 2 Department of Geography, College of Science, National Taiwan University, Taipei, Taiwan; 3 Department of Internal Medicine, National Taiwan University Hospital, Taipei, Taiwan; McGill University, Canada

## Abstract

Tuberculosis (TB) disease can be caused by either recent transmission from infectious patients or reactivation of remote latent infection. Spatial dependency (correlation between nearby geographic areas) in tuberculosis incidence is a signature for chains of recent transmission with geographic diffusion. To understand the contribution of recent transmission in the TB endemic in Taiwan, where reactivation has been assumed to be the predominant mode of pathogenesis, we used spatial regression analysis to examine whether there was spatial dependency between the TB incidence in each township and in its neighbors. A total of 90,661 TB cases from 349 townships in 2003–2008 were included in this analysis. After adjusting for the effects of confounding socioeconomic variables, including the percentages of aboriginals and average household income, the results show that the spatial lag parameter remains positively significant (0.43, p<0.001), which indicates that the TB incidences of neighboring townships had an effect on the TB incidence in each township. Townships with substantial spatial spillover effects were mainly located in the northern, western and eastern parts of Taiwan. Spatial dependency implies that recent transmission plays a significant role in the pathogenesis of TB in Taiwan. Therefore, in addition to the current focus on improving the cure rate under directly observed therapy programs, more resource need to be allocated to active case finding in order to break the chain of transmission.

## Introduction

Human tuberculosis (TB) is an airborne infectious disease caused by *Mycobacterium tuberculosis*. The risk of progressing to active disease is highest in the first 2 years after infection, during which half of the symptomatic TB cases occur [1]. Active TB disease can be the result of either recent transmission from infectious patients or reactivation of remote latent infection [1], [2]. Genotyping and geospatial scanning investigations by the Centers for Disease Control and Prevention (Atlanta, Georgia) have shown that approximately 1 in 4 active TB cases reported in the United States may be attributed to recent transmission [2]. Limited data are available for the role of recent transmission in nationwide TB incidence in other countries.

Taiwan is a middle-burden country with an annual TB incidence remaining around 70 per 100,000 people from 1997 through 2005 [3], despite BCG vaccination and anti-TB drug therapy. National Directly Observed Treatment (DOT) programs were started in 2006, and the annual TB incidence gradually decreased to 57 per 100,000 people in 2010 [3]. Reactivation has been assumed to be the predominant mode of pathogenesis because of age transition in tuberculosis patients – from a disease of young adults during 1957–1961 to a disease of elderly (≥65 years) people during 1997–2001 [4]. Although several outbreaks of active TB cases, which occurred in a family or within a hospital, were identified using genotyping techniques [5]–[9], there remains a lack of nationwide genotyping or geospatial investigations on the role of recent transmission in the TB endemic in Taiwan.

Because the cumulative effect of local TB transmission among communities will cause geographic diffusion, we hypothesize that, if recent transmission plays a significant role in the TB endemic in Taiwan, we should be able to observe the presence of spatial dependency (the correlation between nearby geographical areas) in TB incidence between neighboring townships after adjusting for the spatial autocorrelation of the underlying sociodemographic and ethnic factors that influence the incidence of TB and TB reactivation (i.e. age, economic status, human immunodeficiency virus (HIV) infection, and aborigines) [10], [11].

To understand the role of recent transmission in TB endemic in Taiwan, we applied spatial regression analyses to examine whether spatial dependency exists for the TB incidence at the township-level, after adjusting for the effects of socioeconomic geography.

## Methods

### Data Sources

Pulmonary TB is a notifiable disease that must be reported in Taiwan. Anonymized data on TB cases were obtained from the Notifiable Infectious Disease Statistics System [3] of Taiwan Centers for Disease Control (Taipei, Taiwan). Cases occurring from 2003–2008 were included in this study. The townships where TB cases occurred were mapped according to the patients’ residential addresses. Cases from the outlying islands (including Penghu County, Kinmen County, Lienchiang County, Green Island, Orchid Island, and Liu Chiu Island) were excluded. A total of 90,661 TB cases from 349 townships were included. The TB incidence of each township was estimated using the number of TB cases during a period divided by total population of the township. Demographic and socioeconomic data were obtained from the 2000 Taiwan Census. Anonymized data on HIV infection cases were also obtained from the Notifiable Infectious Disease Statistics System [12].

### Ethics Statement

Taiwan Centers for Disease Control (Taipei, Taiwan) approved the use of data for the present study. The study procedure was reviewed and approved by the Institutional Review Board (IRB) of National Taiwan University Hospital (Taipei, Taiwan). The IRB approved the exemption of informed consent because the data on TB and HIV cases had been anonymized by the Notifiable Infectious Disease Statistics System.

### Socioeconomic Variables

Taiwan Census data included the population density, average household income, average number of persons per household, average years of education, and percentages of the population that were elderly (>60 years), aboriginal, Southeast Asian brides, and Southeast Asian laborers for each township. The average household income and average years of education were analyzed by quartiles using dummy variables (see Table 1 for details).

**Table 1:** Descriptive statistics and univariate regression analyses.

Variable Abbreviation	Definition			Mean (SD)		Regression coefficient†	Regression coefficient††	
TB_INCI		2003–2008 TB cumulative incidence	0.0052 (0.0035)		–		–	
TB_INCI_6		2006–2008 TB cumulative incidence	0.0024 (0.0016)		–		–	
ABOR_P		Aborigines %	0.0775 (0.1966)			**1.60** ***		**1.46** ***
BRIDE_P		% of population of brides from Southeast Asia	0.0001 (0.0002)			**−391.57** ***		**−368.01** ***
DENSITY		Township population/area (m^2^)	0.0029 (0.0061)			−16.08**		−13.11**
EDU1		8.2<Education years< = 8.7 (lower middle)	–			**−**0.06		**−**0.05
EDU2		8.7<Education years< = 9.5 (middle)	–			**−0.12** *		**−**0.09
EDU3		Education years>9.5 (high)	–			**−0.23** ***		−0.19**
ELDER_P		% of Population >60 years old	0.1413 (0.0398)			**−**0.20		**−**0.83
HIV_INCI		1984–2002 HIV cumulative incidence	0.0001 (0.0001)			**410.19** *		**391.30** *
HOU_PERS		Average number of persons per household	3.5027 (0.4262)			0.15**		0.16**
INCOME1		320<Average household income< = 440 (lower middle)	–			0.08		0.06
INCOME2		440<Average household income< = 560 (middle)	–			−0.17**		**−0.13** *
INCOME3		Average household income>560 (high)	–			**−0.42** ***		**−0.36** ***
LABOR_P		% of population of laborers from Southeast Asia	0.0109 (0.0152)			**−6.93** ***		−5.09**

* p<0.05 **p<0.01

*** p<0.001

† Dependent variable: ln (TB_INCI)

†† Dependent variable: ln (TB_INCI_6).

ABOR_P, BRIDE_P, DENSITY, HIV_INCI, and LABORER_P were log transformed in regression analysis.

Average household income (in thousands Taiwan Dollars) was calculated using total income divided by number of households. The percentage of the population who received primary, junior high, senior high, bachelor’s, master’s and doctoral education were given. We used this information to calculate an average years of education for each township by giving a weight of 6, 9, 12, 16, 18 and 22 years to each education level. Because we were interested in how education level would affect the incidence of TB, we classified the average years of education into four groups using the quartiles as cutoff points. In this way, there were three dummy variables with the lowest serving as the reference group. The same procedure was performed for the average household income.

### Spatial Autocorrelation and the Spatial Weight Matrix

Spatial autocorrelation identifies the patterns of spatial dependency by calculating the correlation of a variable with itself within a geographic space, meaning that the value of a variable is associated with those of the same variable in nearby areas. If spatial autocorrelation exists, general statistical methods that assume values of observations are independent may be invalid for further analysis. Spatial autocorrelation can occur in two directions: positive and negative. Positive spatial autocorrelation implies that the values of neighboring areas are similar to one another, while negative autocorrelation implies they are opposed to each other. The statistic used in this study to measure spatial autocorrelation is Moran’s I. This measure is used for variables at interval or ratio scales. The value of Moran’s I is calculated based on the deviation from the mean of two neighboring values [13]. The mathematical formula is as follows:
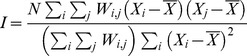
(1)


where *N* is the sample size, 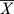 is the mean of the variable, *X_i_* is the value of the variable at a particular location i, *X_j_* is the variable value at location j, and *W_ij_* is a spatial weight indexing the location of i relative to j. The value of this statistic is scored between −1 and 1. A score close to 1 represents positive autocorrelation and townships that may be hot spots. A score near **−**1 shows negative autocorrelation, indicating that the values of neighboring areas are opposite that of the township being examined. The significance of Moran’s I is evaluated by using a Z score and p-value generated by random permutation. The null hypothesis states that there is no spatial autocorrelation for the variable within the geographic area.

Spatial neighbors can be defined by a spatial weight matrix that is created in accordance with the neighbor definition chosen. We first calculated the mean distances between population centers of townships. Townships with shorter distances between their population centers were defined as neighbors. The geospatial relationships between pairs of the 349 townships were stored in a 349×349 matrix. The weight of each cell was the inverse of the distance between the two neighbors.

### Spatial Lag Model

We inspected the residuals from the ordinary least squares (OLS) regression model to identify the spatial dependency of the residuals. If spatial dependency exists, it violates the assumption that the error terms of individual observations are independent of each other in the OLS regression; therefore a model that considers spatial autocorrelation is necessary [14].

We used a spatially lagged y model [15] that incorporated a spatially lagged dependent variable (y) on the right side of the regression equation. This regression model in matrix notation is represented as follows:
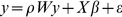
(2)where ρ is the spatial lag parameter, W is the spatial weight matrix, X is a matrix of explanatory variables with an associated vector of regression coefficients β, and ε is a vector of normally distributed, random error terms. When the parameter associated with spatial lag (ρ) was positive, it indicated that, for townships where TB incidence was high, their neighbors also had a high TB incidence.

Because y is recruited in both sides of the regression equation, spatial dynamics creates a feedback effect between townships, in which a township’s level of TB incidence has an effect on its neighbors’, and the neighbors’ neighbors are also affected, throughout all connected townships [14]. This phenomenon leads to a chain reaction that finally returns to influence the initial TB incidence via the spatially lagged y term. In equilibrium, the expected value for y is calculated as follows:
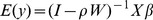
(3)


The spatial multiplier, (I-ρW)^−1^, shows how much the change in independent variable x in one township “spills over” onto other surrounding townships. This “spillover” then affects y through the effect of its spatial lag [16].

We used maps and a histogram to illustrate the variability in the spatial spillover (diffusion) of each township. These figures present the spillover (diffusion) at equilibrium of TB incidence into the surrounding townships with one-unit changes in the explanatory variables.

We were also interested in determining whether a neighbors’ previous TB incidence could be associated with that township’s future TB incidence. The space-time model appears as y_t_ = ρWy_t−1_+Xβ+ε, where we set y_t_ as the TB incidence from 2006–2008 and y_t−1_ as the TB incidence from 2003–2005.

### Statistical Analysis

The associations between socioeconomic variables and TB incidences were analyzed using a linear regression model. Natural logarithmic transformations were used for TB incidence to accommodate the assumption of normal distribution. Stepwise regression modeling was conducted using SAS version 9.2 (SAS Institute, Cary, North Carolina). Moran’s I statistic calculation, the permutation process, and the spatial regression analysis were performed using Geoda® version 0.9.5-I [17]. The spatial multiplier was calculated using R version 2.9.0.

## Results

### Spatial Distribution of TB Incidence

**Figure 1:**
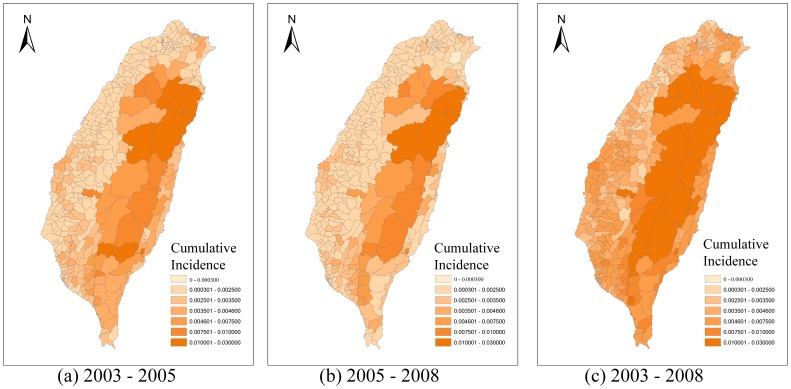
Spatial distribution of the cumulative incidence of TB over different time periods: (a) 2003–2005, (b) 2006–2008, and (c) 2003–2008.

Figure 1 shows the TB incidences of the 349 townships from 2003–2008. The Moran’s I statistic for the TB incidences of the 349 townships was positive (0.37) and statistically significant, indicating the presence of spatial clustering of TB incidences.

### Univariate Analysis and OLS Model

We performed linear regression to identify the socioeconomic variables associated with higher TB incidence. Univariate analyses were performed for each independent variable, and they showed that most socioeconomic variables were significant, except for lower middle education (EDU1), the percentage of elderly (ELDER_P), and lower middle income (INCOME1) (Table 1). There was also a correlation between the socioeconomic variables (Table 2). We subsequently conducted stepwise multiple regression analysis, which showed that only the percentages of aborigines (ABOR_P), middle income (INCOME2), and high income (INCOME3) were independent factors (Table 3). The variance inflation factor of these variables remained below 2, excluding multicollinearity.

**Table 2:** Correlation matrix showing Pearson’s correlation coefficient between socioeconomic variables.

Variables	ABOR_P	BRIDE_P	DENSITY	EDU1	EDU2	EDU3	ELDER_P	HIV_INCI	HOU_PERS	INCOME1	INCOME2		INCOME3		LABOR_P	
TB_INCI	**+0.67** *	**−0.21** *	**−0.21** *	**−**0.05	**−0.11** *	**−0.22** *	**−**0.02	**+0.13** *	**+0.14** *	+0.07	**−0.16** *		**−0.38** *		**−0.23** *	
ABOR_P	1	**−**0.09	**−0.17** *	**−0.16** *	**−0.18** *	**−0.18** *	**−0.17** *	**+0.28** *	**+0.19** *	**−**0.10	**−0.20** *		**−0.20** *		**−0.23** *	
BRIDE_P		1	**+0.28** *	**−0.16** *	**−**0.10	**+0.46** *	**−0.16** *	**+0.27** *	**−0.27** *	**−0.21** *	**−**0.09		**+0.43** *		+0.05	
DENSITY			1	**−0.19** *	**−**0.07	**+0.53** *	**−0.28** *	**+0.44** *	**−0.39** *	**−0.21** *	+0.02		**+0.44** *		+0.00	
EDU1				1	**−0.31** *	**−0.31** *	**+0.18** *	**−0.22** *	**+0.27** *	**+0.21** *	+0.02		**−0.15** *		**−**0.02	
EDU2					1	**−0.32** *	**−0.20** *	**−**0.09	+0.05	+0.08	**+0.25** *		**−0.04** *		+0.11	
EDU3						1	**−0.32** *	**+0.34** *	**−0.47** *	**−0.23** *	+0.01		**+0.53** *		+0.05	
ELDER_P							1	**−0.33** *	**−**0.02	**+0.30** *	**−0.21** *		**−0.32** *		**−0.23** *	
HIV_INCI								1	**−0.31** *	**−0.12** *	**−**0.10		**+0.25** *		**−**0.00	
HOU_PERS									1	+0.09	+0.08		**−0.17** *		**−**0.01	
INCOME1										1	**−0.34** *		**−0.33** *		**−0.13** *	
INCOME2											1		**−0.33** *		**+0.13** *	
INCOME3												1		**+0.24** *		
LABOR_P														1		

* p<0.05 coefficient >0.3 or<**−**0.3.

See Table 1 for variables abbreviation.

We again used Moran’s I statistic to test if there was still spatial autocorrelation for the residuals of OLS regression. The Moran’s I statistic was 0.18, indicating that the independent variables in the OLS model did not account for all spatial dependence in the outcome variable. These results confirmed the need to conduct spatial regression.

### Spatial Lag Model

Spatial lag regression was conducted using the distance between population centers of polygons as the spatial weight. These results are shown in Table 3. Both the percentage of aborigines and high household income remain significant in the spatial lag model. A high percentage of aborigines was associated with higher TB incidence, while a high average household income was associated with lower TB incidence. These associations became smaller in the spatial lag model. Middle income (INCOME2) was significant in the OLS model but not in the spatial lag model. The ρ coefficient for the spatial parameter was significant and positive (0.43, p<0.001), which implies a positive correlation between the TB incidences of neighboring townships.

**Table 3:** Multiple regression analyses: ordinary least square (OLS) model, spatial lag model, and spatial time lag model.

Variable	OLS model^∧^	Spatial Lag model^†^	Spatial-Time Lag model††
ABOR_P	1.38***	1.19***	1.15***
INCOME2	**−**0.15***	**−**0.08	**−**0.07
INCOME3	**−**0.34***	**−**0.21***	**−**0.22***
Spatial Lag (Wy)	–	0.43***	–
Spatial Time Lag (Wy_t−1_)	–	–	64.63**
Adjusted R^2^	0.53	–	0.42
Log likelihood	**−**97.04	**−**78.52	**−**137.21
AIC	202.08	167.05	284.42

* p<0.05

** p<0.01

*** p<0.001

∧† Dependent variable: ln (TB_INCI)

†† Dependent variable: ln (TB_INCI_6).

See Table 1 for variables abbreviation AIC: Akaike’s information criterion.

The log likelihood and Akaike’s information criterion (AIC) showed that the spatial lag model had a better fit than the OLS model. The Moran’s I statistic for the residuals of the spatial lag model was 0.05, which was very close to 0. This demonstrated that the spatial parameter could eliminate the effect of spatial autocorrelation in the regression model.

### Spatial Multiplier

**Figure 2:**
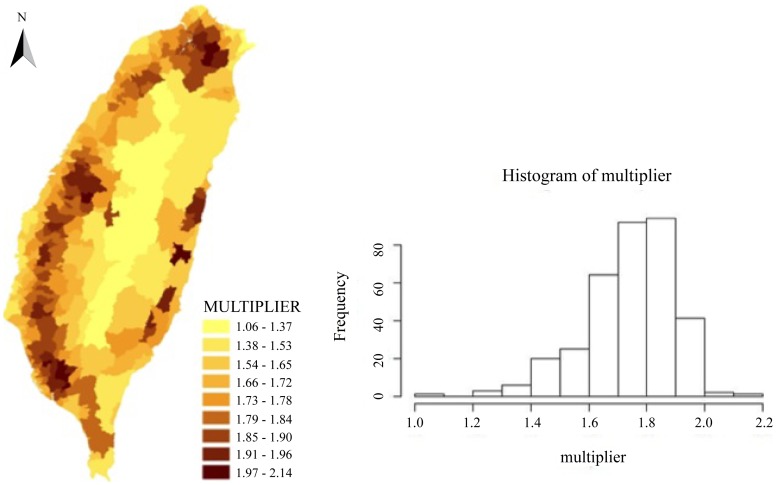
Spatial variations and the histogram of spatial multipliers.

The spatial multiplier for the spatial lag model was calculated for each township and presented in Figure 2. This multiplier represented the interdependence of TB incidence for adjacent townships and had a minimum value of 1.06, which indicated that the independent variables for every township had a certain degree of spillover. The average value was 1.74 and the standard deviation was 0.15. Townships with high spatial multiplier values were mainly located in the northern, western and eastern parts of Taiwan.

### Spatial-Time Lag Model

We further consider a spatial-time lag model. The model appeared as y_t_ = Xβ+ρWy_t−1_+ε, where we set y_t_ as the log-transformed TB incidence from 2006–2008 (under national DOT programs) and y_t−1_ as the log-transformed TB incidence from 2003–2005 (before national DOT programs). Using y_t_ as the dependent variable, univariate analyses were performed; these analyses are presented in Table 1. The result was similar to using the log transformed TB incidence from 2003–2008 as the dependent variable. The term Wy_t−1_ was then calculated along with the percentages of aborigines, middle income, and high income and put into the model as independent variables. The spatial-time lag parameter, percentage of aborigines, and high average household income remained significant (Table 3). Therefore, the neighboring townships’ TB incidences from 2003–2005 were associated with a township’s TB incidence from 2006–2008. Moran’s I for the residuals of the spatial-time lag model (0.13) were much smaller than that of the log transformed TB incidence from 2006–2008 (0.29). Thus, the spatial-time lag parameter could partially eliminate the effect of spatial autocorrelation.

## Discussion

Our geospatial analysis of the countrywide TB data for Taiwan indicated that the TB incidence in a township was significantly affected by the TB incidence in neighboring townships, which implies that recent transmission plays a significant role in TB endemic in Taiwan. Therefore, in addition to the current focus on improving the cure rate under DOT programs, more resource need to be allocated to active case finding in order to break the chain of transmission.

Using spatial regression modeling, we demonstrated that there exists a spatial dependency of township-level TB incidences in Taiwan, after adjusting for the effects of confounding socioeconomic variables, including the percentages of aboriginals and average household income. Furthermore, when we considered the temporality of the infectious processes, the spatial-time lag model indicated that a town’s TB incidences from 2006–2008 were affected by their neighbors’ TB incidences from 2003–2005, as would be expected from the cumulative effects of local TB transmission with contagious diffusion among the community.

The geospatial findings in the present study are consistent with molecular epidemiologic findings [5]–[9]. A large nosocomial TB outbreak in 2003 involving 66 health care workers at a district hospital in Taipei was traced to an index case who was hospitalized in February 2002 by matched DNA fingerprints [5]. Mycobacterial interspersed repetitive-unit-variable-number tandem-repeat (MIRU-VNTR) typing and spoligotyping was performed on TB isolates from 365 patients treated at a hospital in Taipei from 2002–2004; these results showed that 236 (65%) were clustered by genotype [7]. Another study in Hualien County in eastern Taiwan showed that 45 (62%) of 73 multidrug-resistant TB isolates were clustered [9]. These clustering rates were significantly higher than those reported in San Francisco (40%) [18], New York (37.5%) [19], the Netherlands (46%) [20], and Denmark (57%) [21], but lower than those reported from Malawi (72%) [22] and South Africa (67% [23], 72% [24]). Molecular genotyping further revealed that at least 51% of recurrent TB cases in Taiwan were caused by re-infection by a different strain, rather than by relapse or re-activation [25]. In keeping with previous molecular epidemiologic findings, our geospatial analyses provide necessary, complementary evidence on the significant role of recent TB transmission in Taiwan.

Our analysis showed that the percentage of aborigines is an independent risk factor for higher TB incidence after adjusting for the effects of spatial dependency and household income. This finding was in agreement with previous studies of TB incidence in Taiwan [26]–[29]. It has been shown that aboriginal areas have a TB incidence that is 3–5 times higher than non-aboriginal areas [26] and that the socioeconomic and health statuses of people living in aboriginal areas were generally lower than the national average [27], [28]. Poor compliance with anti-TB treatment might lengthen infectious period, thus increasing transmission [29].

Consistent with previous observations that TB is a disease of the deprived and the poor [30]–[32], our analysis found that a high average household income (the highest quartile) is an independent factor for lower TB incidence. TB is related to poverty in a number of ways, including higher contact rates due to crowded and poorly ventilated environments, reduced immunity status, and decreased odds of receiving proper treatment [30]. From Table 2, we can see that high average household income (INCOME3) was significantly correlated with all other variables, which may be the reason that most other socioeconomic variables were insignificant in the stepwise multiple regression analysis.

HIV infection weakens the immunity of patients and increases the risk of rapid progression to active TB disease after infection [33]. High HIV prevalence in the population may increase TB incidence [33]–[35]. In univariate analysis, we found that the cumulative HIV incidence from 1984–2002 was a significant risk factor for higher TB incidences in 2003–2005, as well as 2003–2008 (Table 1). Nevertheless, HIV infection status did not remain an independent factor in the multiple regression model. One probable reason for this change is the low HIV prevalence in Taiwan: there were only 4,145 adult HIV cases at the end of 2002 out of a population of 23 million. In addition, the positive correlation between HIV and high average income (Table 2) could mask the potential effect of HIV infection on TB incidence.

The resolution of the geospatial analysis in the present study was limited to the township level because further details on the residential addresses of TB patients were kept confidential by the Notifiable Infectious Disease Statistics System. Therefore, we were unable to use spatial point analysis methods to identify localized spatial clustering of TB cases. Another limitation of this study is the lack of data on the molecular genotype of clinical isolates and the host factors of individual persons, as well as the social network data, which restricts our inferences to the ecological level. The last limitation is that, if a spatially autocorrelated determinant of reactivated latent TB cases has been overlooked, our conclusions could be incorrect. We do take into consideration a range of important socioeconomic factors, but it is still possible that an important variable is missing. Our findings justify further large-scale genotyping-geospatial correlation studies to provide more insight on TB epidemiology in Taiwan.

In conclusion, our results add to the evidence that recent transmission plays a significant role in TB incidence in Taiwan, as well as highlighting the importance of taking a geospatial perspective in TB epidemiology.
